# Supplementary material for: Interaction between Discs large and Pins/LGN/GPSM2: a comparison across species
Source: Biol Open. 2021 Nov 3;10(11):bio058982. doi: 10.1242/bio.058982 (PMC8576264; doi:10.1242/bio.058982)
Supplement: Supplementary information [file biolopen-10-058982-s1.pdf]

A Dlg

	H. sapiens	M. musculus	X. laevis	D. rerio	S. purpuratus	D. melanogaster	C. gigas	C. elegans	E. diaphana	A. tenebrosa	N. vectensis	T. adhaerens	O. lobularis	O. minuta	Average
H. sapiens	100	99.59	87.43	84.33	59.29	55.34	59.83	44.57	59.34	57.23	58.82	49.5	54.65	31.9	61.68%
M. musculus	99.59	100	87.71	84.05	59.29	55.34	59.68	44.57	59.34	57.23	58.82	49.36	54.8	32.06	61.68%
X. laevis	87.43	87.71	100	80.35	60.95	53.39	57.73	42.98	59.12	54.87	56.47	46.57	52.65	32.42	59.43%
D. rerio	84.33	84.05	80.35	100	59.42	50.85	56.12	41.41	58.61	55.39	55.86	45.05	50.97	32.43	58.06%
S. purpuratus	59.29	59.29	60.95	59.42	100	55.51	61.55	44.72	62.3	62.5	62.39	48.66	55.32	33.28	55.78%
D. melanogaster	55.34	55.34	53.39	50.85	55.51	100	63.15	42.81	57.26	57.28	55.63	42.56	52.65	33.42	51.94%
C. gigas	59.83	59.68	57.73	56.12	61.55	63.15	100	43.97	62	61.17	61.71	46.64	57.72	33.38	55.74%
C. elegans	44.57	44.57	42.98	41.41	44.72	42.81	43.97	100	45.34	42.91	44.5	35.68	42.23	30.82	42.04%
E. diaphana	59.34	59.34	59.12	58.61	62.3	57.26	62	45.34	100	85.02	80.73	51.57	58.35	33.54	59.42%
A. tenebrosa	57.23	57.23	54.87	55.39	62.5	57.28	61.17	42.91	85.02	100	79.02	45.94	58.41	33.75	57.75%
N. vectensis	58.82	58.82	56.47	55.86	62.39	55.63	61.71	44.5	80.73	79.02	100	45.87	59.04	34.5	57.95%
T. adhaerens	49.5	49.36	46.57	45.05	48.66	42.56	46.64	35.68	51.57	45.94	45.87	100	42.17	29.93	44.58%
O. lobularis	54.65	54.8	52.65	50.97	55.32	52.65	57.72	42.23	58.35	58.41	59.04	42.17	100	34.33	51.79%
O. minuta	31.9	32.06	32.42	32.43	33.28	33.42	33.38	30.82	33.54	33.75	34.5	29.93	34.33	100	32.75%

B Dlg-GUK

	H. sapiens	M. musculus	X. laevis	D. rerio	S. purpuratus	D. melanogaster	C. gigas	C. elegans	E. diaphana	A. tenebrosa	N. vectensis	T. adhaerens	A. queenslandica	O. lobularis	O. minuta	M. brevicollis	S. rosetta	Average
H. sapiens	100	100	93.33	93.33	76.67	86.67	80	61.02	76.67	73.33	75	66.67	73.33	72.88	54.24	59.32	59.32	75.11%
M. musculus	100	100	93.33	93.33	76.67	86.67	80	61.02	76.67	73.33	75	66.67	73.33	72.88	54.24	59.32	59.32	75.11%
X. laevis	93.33	93.33	100	91.67	78.33	86.67	81.67	61.02	78.33	75	76.67	66.67	73.33	72.88	52.54	59.32	57.63	74.91%
D. rerio	93.33	93.33	91.67	100	81.67	91.67	83.33	64.41	78.33	75	76.67	66.67	75	76.27	54.24	59.32	57.63	76.16%
S. purpuratus	76.67	76.67	78.33	81.67	100	85	88.33	59.32	75	81.67	78.33	68.33	73.33	72.88	50.85	61.02	54.24	72.60%
D. melanogaster	86.67	86.67	86.67	91.67	85	100	90	66.1	83.33	78.33	81.67	65	80	79.66	55.93	57.63	57.63	77.00%
C. gigas	80	80	81.67	83.33	88.33	90	100	61.02	83.33	85	85	70	75	76.27	52.54	57.63	54.24	75.21%
C. elegans	61.02	61.02	61.02	64.41	59.32	66.1	61.02	100	57.63	54.24	55.93	59.32	62.71	61.02	58.62	52.54	55.93	59.49%
E. diaphana	76.67	76.67	78.33	78.33	75	83.33	83.33	57.63	100	90	90	65	70	77.97	54.24	55.93	50.85	72.71%
A. tenebrosa	73.33	73.33	75	75	81.67	78.33	85	54.24	90	100	90	68.33	66.67	76.27	50.85	55.93	49.15	71.44%
N. vectensis	75	75	76.67	76.67	78.33	81.67	85	55.93	90	90	100	70	68.33	72.88	52.54	57.63	52.54	72.39%
T. adhaerens	66.67	66.67	66.67	66.67	68.33	65	70	59.32	65	68.33	70	100	58.33	67.8	55.93	55.93	57.63	64.27%
A. queenslandica	73.33	73.33	73.33	75	73.33	80	75	62.71	70	66.67	68.33	58.33	100	69.49	50.85	55.93	52.54	67.39%
O. lobularis	72.88	72.88	72.88	76.27	72.88	79.66	76.27	61.02	77.97	76.27	72.88	67.8	69.49	100	55.17	54.24	54.24	69.55%
O. minuta	54.24	54.24	52.54	54.24	50.85	55.93	52.54	58.62	54.24	50.85	52.54	55.93	50.85	55.17	100	44.83	46.55	52.76%
M. brevicollis	59.32	59.32	59.32	59.32	61.02	57.63	57.63	52.54	55.93	55.93	57.63	55.93	55.93	54.24	44.83	100	62.71	56.82%
S. rosetta	59.32	59.32	57.63	57.63	54.24	57.63	54.24	55.93	50.85	49.15	52.54	57.63	52.54	54.24	46.55	62.71	100	55.13%

C Khc73-MBS

	H. sapiens	M. musculus	X. laevis	D. rerio	D. melanogaster	C. gigas	E. diaphana	A. tenebrosa	N. vectensis	A. queenslandica	Average
H. sapiens	100	95.9	81.15	76.23	49.58	50.82	46.96	46.09	45.22	37.84	58.86%
M. musculus	95.9	100	81.15	76.23	49.58	50	48.7	46.96	45.22	36.94	58.96%
X. laevis	81.15	81.15	100	76.23	51.26	48.36	47.83	45.22	46.09	34.23	56.84%
D. rerio	76.23	76.23	76.23	100	52.1	44.26	42.61	41.74	38.26	31.53	53.24%
D. melanogaster	49.58	49.58	51.26	52.1	100	60.33	47.79	46.9	45.13	38.18	48.98%
C. gigas	50.82	50	48.36	44.26	60.33	100	52.99	54.7	51.28	36.75	49.94%
E. diaphana	46.96	48.7	47.83	42.61	47.79	52.99	100	84.55	81.3	43.75	55.16%
A. tenebrosa	46.09	46.96	45.22	41.74	46.9	54.7	84.55	100	83.87	42.86	54.77%
N. vectensis	45.22	45.22	46.09	38.26	45.13	51.28	81.3	83.87	100	44.64	53.45%
A. queenslandica	37.84	36.94	34.23	31.53	38.18	36.75	43.75	42.86	44.64	100	38.52%

Percent Identity

- 100%
- 80-99.99%
- 60-79.99%
- 40-59.99%
- 20-39.99%
- 0-19.99%

D GPSM2

	H.sapiens	M.musculus	X.laevis	D.rerio	S.purpuratus	D.melanogaster	C.gigas	C.elegans GPR1	C.elegans GPR2	E.diaphana	A.tenebrosa	N.vectensis	T.adhaerens	A.queenslandica	Average
H.sapiens	100	92.32	79.45	71.86	56.24	53	58.78	18.12	18.55	56.1	57.91	57.09	49.11	32.59	53.93%
M.musculus	92.32	100	78.78	71.63	55.7	53.25	58.68	17.23	17.66	55.75	57.12	56.04	49.02	32.66	53.53%
X.laevis	79.45	78.78	100	70.97	57.19	56.06	60.74	15.74	16.17	57.19	58.32	57.6	50.27	31.93	53.12%
D.rerio	71.86	71.63	70.97	100	55.24	55.42	57.96	19.59	20	55.61	56.31	56.37	48.7	31.11	51.60%
S.purpuratus	56.24	55.7	57.19	55.24	100	50.69	59.34	17.4	17.62	58.97	59.15	60.45	49.28	28.91	48.17%
D.melanogaster	53	53.25	56.06	55.42	50.69	100	54.73	15.35	15.58	53.6	53.97	53.87	44.36	29.69	45.35%
C.gigas	58.78	58.68	60.74	57.96	59.34	54.73	100	16.55	16.78	60.1	60.97	60.67	49.82	29.2	49.56%
C.elegans (GPR 1)	18.12	17.23	15.74	19.59	17.4	15.35	16.55	100	96.95	15.42	15.85	15.84	17.75	16.15	22.92%
C.elegans (GPR 2)	18.55	17.66	16.17	20	17.62	15.58	16.78	96.95	100	15.42	15.85	15.84	17.03	16.43	23.07%
E.diaphana	56.1	55.75	57.19	55.61	58.97	53.6	60.1	15.42	15.42	100	85.81	82.74	51.83	28.73	52.10%
A.tenebrosa	57.91	57.12	58.32	56.31	59.15	53.97	60.97	15.85	15.85	85.81	100	81.88	52.29	29.44	52.68%
N.vectensis	57.09	56.04	57.6	56.37	60.45	53.87	60.67	15.84	15.84	82.74	81.88	100	53.89	29.22	52.42%
T.adhaerens	49.11	49.02	50.27	48.7	49.28	44.36	49.82	17.75	17.03	51.83	52.29	53.89	100	26.61	43.07%
A.queenslandica	32.59	32.66	31.93	31.11	28.91	29.69	29.2	16.15	16.43	28.73	29.44	29.22	26.61	100	27.90%

Fig. S1. Percent Identity Matrices. Percent identity matrices for **A)** full length Dlg; **B)** the Dlg-GUK domain; **C)** the MAGUK-binding stalk of Khc73; and **D)** full length Pins/LGN/GPSM2.

Biology Open • Supplementary information

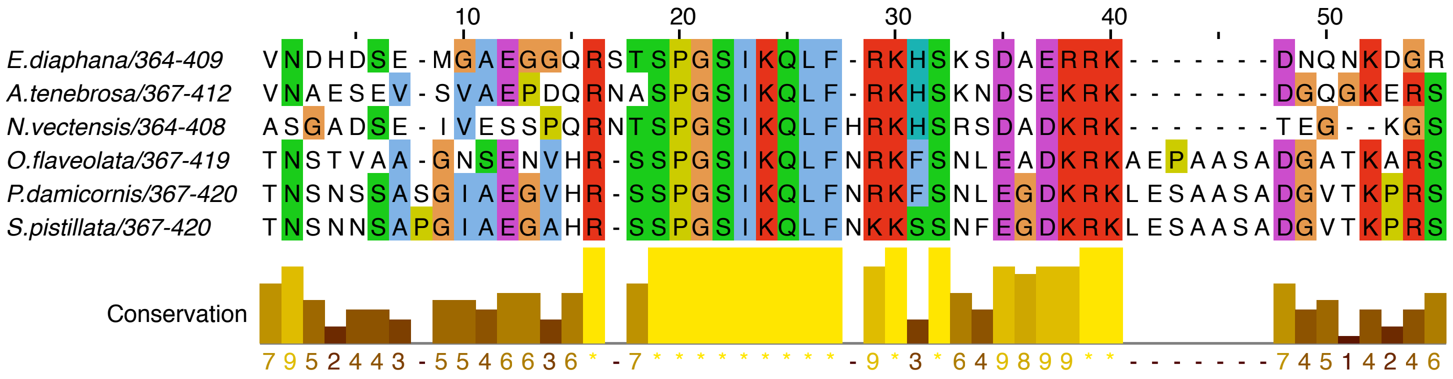

Fig. S2. The Cnidarian Pins/LGN/GPSM2 linker. Sequence alignment at the Pins/LGN/GPSM2 linker region in Cnidaria. Alignment is colored using ClustalX color scheme. Figure is adapted from Jalview 2 (Waterhouse et al., 2009).

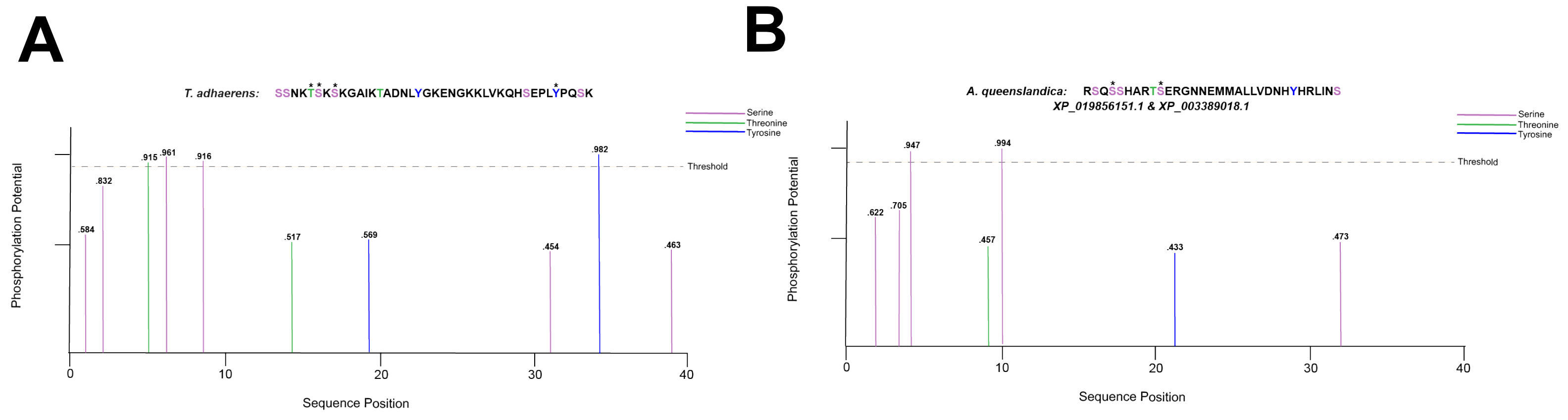

C

complement	NW_003546593.1 Exons
10711...10795	MAETGISVHS RMMAQDIFRI LSIAFIKEX
10901...11001	LVCFKTRQPL ACSVLNGLTE PSANVSLSY
12193...12217	IMRTPLGYLY NKDVCPNWYK PI
12593...12642	RDYSLRKTAN GGYYQYX
12781...12966	PFVKLRKXMS VCGPRIIRCR EYXHSKHCMG SMDTPTSYKT SKLTQTQLAS QPQLTHQXXL EL
13327...13360	SCGREVRRT
13424...13473	GTSIFRKKSI PLHVT
14350...14412	FCPVLIYLNK FLSSTLDVLF Y

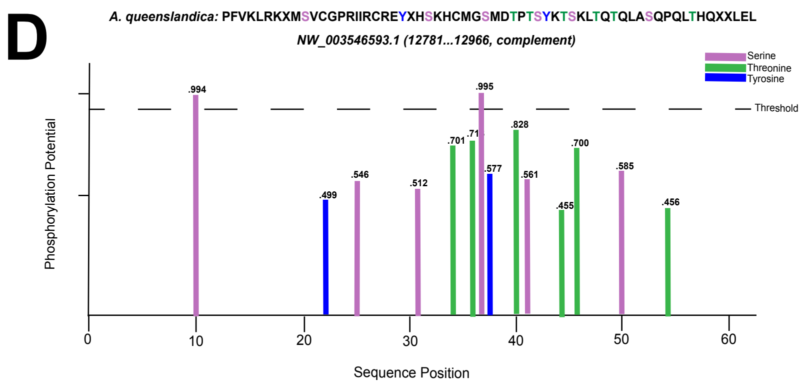

Fig. S3. Phosphorylation site predictions. Predicted phosphorylation potential of **A)** T. adhaerens (Placozoa) and **B)** A. queenslandica (Porifera) Pins/LGN/GPSM2 linker regions. Asterisks indicate the residues that are predicted to be a phosphorylation site with greater than 90% confidence. Graphs are adapted from NetPhos 3.1 (Blom et al., 1999). **C)** Reverse translated nucleotide sequences of eight regions with high exon character between NW_003546593.1 (14479...19908, complement) and NW_003546593.1 (9550…10710, complement). **D)** Of the eight potential exons identified, only one had predicted phosphorylation sites.

Biology Open • Supplementary information
